# Sensor Fusion with NARX Neural Network to Predict the Mass Flow in a Sugarcane Harvester

**DOI:** 10.3390/s21134530

**Published:** 2021-07-01

**Authors:** Jeovano de Jesus Alves de Lima, Leonardo Felipe Maldaner, José Paulo Molin

**Affiliations:** 1 Precision Agriculture Laboratory, Biosystems Engineering Department, ‘Luiz de Queiroz’ College of Agriculture, University of São Paulo, Av. Pádua Dias 11, Piracicaba 13418-900, São Paulo, Brazil; jeovanolima@gmail.com (J.d.J.A.d.L.); leonardofm@usp.br (L.F.M.); 2 AGCO Corporation, Av. Guilherme Schell, 10260, Canoas 92420-000, Rio Grande do Sul, Brazil

**Keywords:** precision agriculture, artificial intelligence, machine automation, yield monitor, self-calibration, data fusion

## Abstract

Measuring the mass flow of sugarcane in real-time is essential for harvester automation and crop monitoring. Data integration from multiple sensors should be an alternative to receive more reliable, accurate, and valuable predictions than data delivered by a single sensor. In this sense, the objective was to evaluate if the fusion of different sensors installed in a sugarcane harvester improves the mass flow prediction accuracy. A harvester was experimentally instrumented, and neural network models integrated sensor data along the harvester to perform the self-calibration of these sensors and estimate the mass flow. Nonlinear autoregressive networks with exogenous input (NARX) and multiple linear regression (MLR) models were compared to predict the mass flow. The prediction with the NARX showed a significant superiority over MLR. MLR decreases the estimated mass flow variability in the harvester. NARX with multi-sensor data has an RMSE of 0.3 kg s^−1^, representing a MAPE of 0.7%. The fusion of sensor signals improves prediction accuracy, with higher performance than studies with approaches that used a single sensor. The mass flow approach with multiple sensors is a potential approach to replace conventional yield monitors. The system generates accurate data with high sample density within sugarcane rows.

## 1. Introduction

Mass flow is an essential parameter for the automation of the control processes performed by the harvester. These data support engine rotation control, harvester speed, clean system rotation, chopper blade rotation, and feeder roll speed. The automation of these systems can bring benefits such as a decrease in the damage to the billets [1], uniform billet lengths [2], quality of cleaning, and reduction of losses of the harvested product [3,4]. The mass flow predicted in the harvester provides data necessary for the localized management of sugarcane plantations. Through the mass flow predicted, it is possible to calculate the yield in a given area (e.g., ton ha^−1^), and together with the coordinates, yield maps can be generated. Yield maps are the key to managing sugarcane spatial variability [5,6]. This is the starting point for implementing Precision Agriculture (PA) practices, which aim to assist decision-making and interventions in high spatial definition in the agricultural fields.

Different sugarcane yield monitors have been taken to predict the sugarcane mass being harvested. The first approach to measure the mass flow for mapping sugarcane yield was performed by [7,8]. From then on, yield monitors using load cells [9], optical fiber [10], deflection plates [11], and 3D sensors [12] have already been tested on harvesters to measure the amount of sugarcane processed in real-time. However, according to [13], few are being used commercially due to their accuracy, the continual need for calibration, and the fact that most were developed for research purposes.

In a recent study, the prediction of sugarcane yield using four variables of the harvester engine shows promising results, with fewer minor prediction errors than studies with a single sensor approach [14]. In this sense, we hypothesize that the fusion of data generated by sensors installed in the recollection may be an alternative to reduce the error in mass flow prediction. Sensor fusions have the advantage of making information more significant and precise than a single sensor, and can reduce sensor errors [15]. In agriculture, the integration of the different sensor data is used to improve the autonomous navigation of mobile robots [16], and increase the accuracy of the guide-line of an autonomous robot for the weeding operation [17]. In the study by [18], data coming from heterogeneous but complementary sensors were combined to generate a multi-layer map of the environment and the supporting ground.

Among the models used for sensor data fusion, artificial neural networks (ANN) provide a new approach to the problem of parameter estimation of linear and non-linear models [19]. The learning ability of ANN allows them to adjust to changing, dynamic environments, and is a more flexible predictive tool than traditional statistical models [20,21]. The nonlinear autoregressive networks with exogenous input (NARX) differ from the other ANN in that the outputs of the model are feedback as an input for future predictions. NARX has the advantage of not requiring many different input variables, and the integration of multiple variable inputs and autoregressive inputs benefits the modeling process to provide more accurate estimates [22]. NARX was used to predict and compensate the inertial navigation system position errors when global positioning system (GPS) is unavailable [23,24], diagnose faults for pneumatic control valves [25], predict marine engine performance parameters [26], underwater passive target state estimation [27], undergo multiple data fusion for rainfall estimation [28,29], and perform long-term machine state forecasting [21].

On the other hand, there are still no reports on the use of the NARX to process signals from sugarcane harvester sensors. Thus, this study makes the first approach to investigate the use of the NARX on harvested sugarcane. The NARX can incorporate the dynamics of signals from sensors [25], such as those installed in sugarcane harvesters that monitor the harvester’s operational processes. In this way, the use of the data generated by the sensors already installed in the harvester and harvester instrumentation to measure sugarcane parameters during harvest would be an essential advancement in harvester automation and agricultural operations [30].

The use of the fusion techniques with the sensors in the sugarcane harvester to obtain a single output for the flow mass monitor can be an option to improve the accuracy of prediction. Embedded sensors in the sugarcane harvester can collect high resolution data to guide site-specific management within each sugarcane row with high resolution [30]. However, there is still no knowledge about the use of a combination of hydraulic pressure, strain gauge, and potentiometric displacement sensors on a single harvester for sugarcane mass flow prediction. Our goal was to test machine learning based on a neural network model to integrate sensors installed at various points in a sugarcane harvester. Moreover, the objective was to evaluate if the fusion of different sensors installed in a sugarcane harvester improves the mass flow prediction accuracy.

## 2. Materials and Methods

### 2.1. Mass Flow Prediction

**Figure 1:**
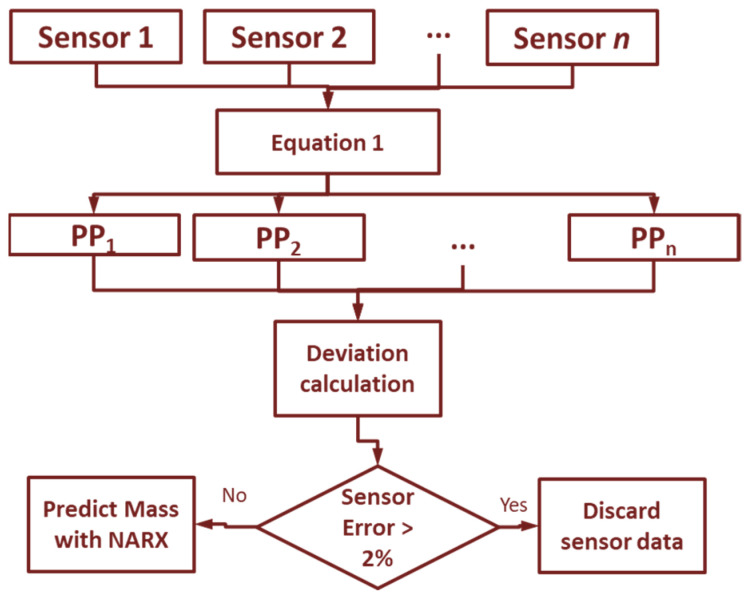
Flowchart of mass flow prediction.

The approach to predicting mass flow in this study is based on sensor signals as input data for models generated through machine learning (ML) (Figure 1). First, the calibration curve was created for the *n* sensor installed on the harvester. The model generated in the initial calibration was used to perform the first preliminary prediction (*PP_n_*) of the mass flow. Due to the characteristics of the sensors and the harvester mechanical factors, it is necessary to recalibrate the sensors over time. Thus, an ML model was used in the *n* sensor to perform the self-calibration, maintaining the accuracy in predicting the mass flow (*PPC_n_*) over the harvester’s operation time. Finally, the fusion of the *PPC_n_* from the n sensor was performed using a supervised model to predict the mass flow in the harvester in real-time. Data processing and model training was performed offline for later use in the harvester.

#### 2.1.1. Sensor Calibration

The calibration curve was obtained by fitting a linear response function (Equation (1)) to a set of experimental data consisting of the sensor measured signal relative to a known ground truth [31].

*y* = *β*_0_ + *β*_1_*x* (1)

where *y* is the ground-truth flow mass value, *x* represents measured sensor signal, and *β*_0_, and gain *β*_1_ are the calibration coefficients. The constant *β*_0_ is the offset that arises when the measured value differs from the true value, and it can be determined by measuring the sensed value when the ground-truth value is zero. Gain *β*_1_ refers to the rate or the amount of change of the measured value to the change in the underlying ground-truth value [32].

#### 2.1.2. Sensor Self-Calibration Approach

To ensure a good precision of a measurement made by the sensors, it is necessary to recalibrate the sensors installed in the harvester. For each sensor, the calibration curve is applied (Equation (1)), generating a preliminary prediction (*PP_n_*). After the *PP* for each n sensor, the mean of the values (*Mppn*—Equation (2)) and the standard deviation (*SDppn*—Equation (3)) are calculated. In this way, it is possible to calculate the prior error of each sensor. If the calculated error (Equation (4)) of sensor *n* is greater than 2%, the data is discarded, and not used for yield prediction. If the sensor keeps registering errors greater than 2% over 10 cycles (10 data collected by the sensor), a warning message is reported. In this way, it is possible to detect a sensor with problems and perform maintenance. In this study, several sensor configurations were tested so that if one of the sensors is defective it is possible to use a prediction model without this sensor, not affecting the yield prediction.

(2) $$Mppn=\sqrt{\frac{1}{n}\overset{n}{\underset{i=1}{\sum }}PP_{n}}$$

(3) $$SDppn=\sqrt{\frac{1}{n}\overset{n}{\underset{i=1}{\sum }}{\left(PP_{n}-Mppn\right)}^{2}}$$

(4) $$Error_{n}\;\left(\%\right)=\left[\frac{PP_{n}}{\left(Mppn\;\pm SDppn\right)}\right]\times 100$$

where *Mppn* is the mean and *SDppn* is the standard deviation of the preliminary prediction (*PP_n_*) of the *n* sensor.

#### 2.1.3. Sensor Fusion

We combine the *PP_n_* from the n sensor into a common MS*_f_* predicted in the stage. To merge the multiples *PP_n_*, machine-learning approaches were used to achieve one fused output. The MLR was used because all commercial sensors for sugarcane flow measure use a linear approach, and NARX was used due to the algorithm having good adaptation with multivariable and nonlinear data. The accuracy of predictions using data fusion methods was compared with those of individual sensors, and among these data fusion methods.

##### NARX Neural Network

**Figure 2:**
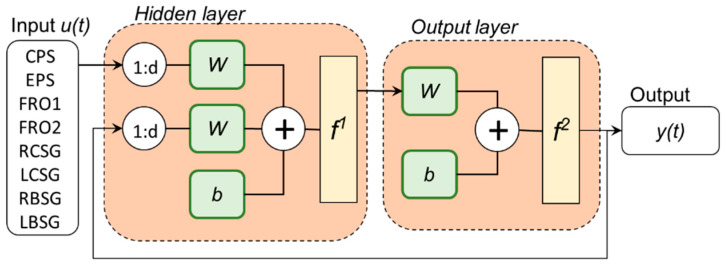
NARX neural network structure. *d* is order delay. *W* is the network input vector and the feedback vector weight matrix. *b* is bias. *f*_1_ is the neural network hidden layer activation function. *f*_2_ is the neural network output layer activation function [35].

The nonlinear autoregressive network with exogenous input (NARX) [33] was chosen due to the excellent ability of this neural network to have good adaptation with multivariable and nonlinear data [34]. A typical NARX regression neural network consists of three layers (input, hidden, and output layer), input and output delay, and the number of hidden layer neurons before application [35] (Figure 2).

The NARX neural network model is a nonlinear autoregressive model that has exogenous inputs. That is, the model relates the current *PP_n_* value of each sensor used in the model, and the value predicted by the model using previous *PP_n_* data. In addition, the model contains an “error” term that relates to the fact that knowledge of other terms will not allow the present value of mass flow to be accurately predicted. The NARX was developed using the Neural Network Toolbox of MATLAB R2018b (MathWorks, Natick, MA, USA) and was calculated as:

*y*(*t*) = *f*(*y*(*t* − 1), *y*(*t* − 2), …, *y*(*t* − *n_y_*), *u*(*t* − 1), *u*(*t* − 2),…, *u*(*t* − *n_u_*)) (5)

where the next value of the dependent output signal *y*(*t*) is regressed on previous values (*y*) of the output signal and previous values of an independent input (*u*) [36]. The function *f* in Equation (5) was trained by the network, which depends on the number of neurons in the hidden layer. In this first study, we used only a hidden layer in the NARX. To find a set of the model’s parameters to produce the best performance, four different numbers of neurons (*m* = 10, 15, 20, and 25) and three order delays (*d* = 3, 5, and 10) were tested. The absolute error between the NARX output and the mass flow value observed was used as the performance measure. The optimal parameters for the NARX were *m* = 15 and *d* = 3. These values obtained the best performance of the model in predicting the sugarcane mass flow.

##### Multiple Linear Regression

The Multiple Linear Regression (MLR) is a parametric model and a supervised learning algorithm that uses a linear approach between the variables. It is the most widely used method to establish relationships between a single dependent and small set of independent variables (Equation (6)).

*y_f_* = *β*_0_ + *β*_1_*x*_*i*1_ + *β*_2_*x*_*i*2_ + … + *β_n_x_in_* + ε (6)

where *y_f_* is the dependent variable (mass flow, kg s^−1^), *x_i_* is exploratory variables, *β*_0_ is the y-intercept (constant term), *β_n_* is the slope coefficients for each exploratory variable deduced from the training dataset, and ε is the model’s error term. The exploratory variables considered were *PPC_n_* from the *n* sensor.

### 2.2. Harvester Instrumentation

**Figure 3:**
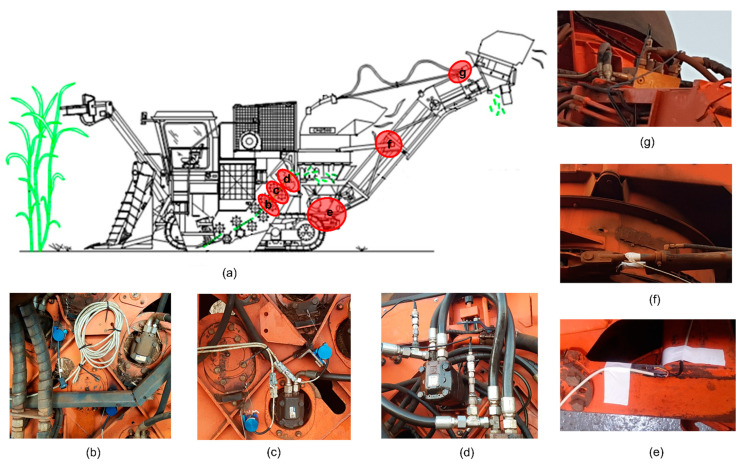
Sensor locations in the harvester (**a**). Potentiometric displacement sensor in the roll feeder number 1 (**b**) and roll feed number 2 (**c**). The pressure sensor in the hydraulic motor of the chopper (**d**). Strain gauges in the elevator base (**e**) and the hydraulic cylinder of the elevator (**f**). The pressure sensor in the hydraulic motor of the elevator (**g**).

A sugarcane harvester, model Valtra BE1035 (AGCO Corporation, Mogi das Cruzes, SP, Brazil), was instrumented with three sensors with different operating principles to predict the mass flow (Figure 3). The sensors were installed in eight different mechanisms throughout the sugarcane flow in the harvester. These mechanisms carry out the processing and transport of sugarcane or are related in some way to the sugarcane flow. Previous researchers on sugarcane yield prediction have already studied the sensors used individually in this study [8,37]. Until this moment, only the scale installed at the end of the elevator can measure the mass flow as a direct response; however, the scale system presents problems such as tilt compensation, vibration, and tare change caused by mineral material, sediment, and debris. [10] use optical sensors to predict sugarcane mass flow in a harvest. Another approach was the installation of instrumentation to monitor the opening of the feeder rollers made by [38,39]. The measurement errors obtained in the reported tests are still about 100% higher than those obtained on grain mass flow used on combines and forage harvesters. In addition, there are reports of only short tests, usually with a calibration adjusted for a full over-flow load. There are no reports of continuous measurement so that the actual behavior of the sensors can be assessed. In this way, we start from the premise that the sensors and the place where they were installed have the potential to predict the mass flow in the harvester.

#### 2.2.1. Pressure Sensor

The pressure sensor, model SCP01-400-14 (Parker Hannifin Corporation, Cleveland, OH, USA), was used to measure the hydraulic pressure in the chopper motor (CEP) and elevator conveyor motor (EPS). This sensor has a reading range from 0 to 400 bar with a response time of ≤1.0 ms. Its non-linearity and hysteresis error was specified as ±0.1% at full scale. This study assumes that the differential hydraulic pressure (bar) in the elevator hydraulic motor is proportional to the amount of sugarcane transported on the elevator conveyor. Also, we consider that differential hydraulic pressure (bar) in the chopper motor is proportional to the amount of sugarcane being chopped.

#### 2.2.2. Strain Gauges Sensor

Unidirectional strain gauges, model P1L11-5/350 (Exel Sensores, Taboao da Serra, SP, Brazil), were used to measure the deformation of elements in the elevator. The strain gauge grid had an area of 1.0 mm^2^, electrical resistance of 350 Ω, and a gauge factor of 2.18. Strain gauges are commonly used to measure mechanical strain and are applied to load cells, torque, and pressure sensors. A finite element model was used to define the strain gauge installation points. Based on this model, it was possible to determine which regions in the elevator would be most sensitive to small sugarcane flow mass variations. The strain gauges were installed in four points in the elevator: left base (LBSG), right base (RBSG), right hydraulic cylinder (LCSG), and left hydraulic cylinder (RCSG). At each point, the strain gauges were configured as a full Wheatstone bridge.

#### 2.2.3. Displacement Sensor

A potentiometric displacement sensor, model WPS-1000-MK46 (Micro Epsilon America, Raleigh, NC, USA), was used to measure the gap between two feed rollers. This sensor has a reading range from 0 mm to 350 mm. Its nonlinearity and hysteresis error was specified as ±0.1% at full scale. This study assumes that the separation of the feed rollers is proportional to the volumetric flow rate. In the feed rolls, the sugarcane bundle has already spread over the entire width of the rollers, allowing for the uniform processing of the sugarcane in the chopper. The sugarcane flow speed in the last set of feed rollers is constant, as the chopper rollers pull the bundle at a uniform rate to obtain a constant stalk length.

### 2.3. Data Collection

The study field is located in Santa Lucia, SP, Brazil (21°41′13.2″ S 48°05′29.3″ W). The variety is RB985879, spaced 1.5 m between the rows. The harvest was performed at an average speed of 1.5 m s^−1^. The data was collected using a harvester that processes only a single row of sugarcane. A data logger with analog inputs was used to connect the sensors. A script was written in LabVIEW software to control the start and end of data acquisition and records the sensor signal data in a text file. The data collection frequency of the pressure sensor was 10 Hz; strain gauge and potentiometric sensors were 400 Hz.

An external sensor was used to measure the mass flow in real-time to train the predictive models and compare the predicted values. This data was used as a dataset to train and test the NARX. Load cells in the infield wagon were installed to measure the amount of sugarcane mass dumped into the infield wagon by the harvester. The load cells in the infield wagon have an independent system for mass acquisition at an acquisition frequency of 10 Hz. Mass data was collected using a timing trigger to evaluate the input time until the mass flow output time. Sensor data and infield wagon data were collected during the harvest of 9 sugarcane rows with 600 m length, giving the data set necessary to train and compare the prediction models proposed in this study (Table 1).

To validate the best models developed, data was collected on a commercial sugarcane field of 49 ha. The instrumented harvester was used to predict the sugarcane yield and the infield wagon was instrumented with load cells to generate the observed data. The data generated by the harvester and the instrumented infield wagon were georeferenced using a Global Navigation Satellite System (GNSS).

### 2.4. Pre-Processing and Training Models

In the first process, null values and global outliers were excluded. Values out of the measurement scale of each sensor were excluded. The global filtering method for identifying outliers was the Interquartile Range (IQR) [40]. After filtering processes, a time offset was applied for each sensor to correct the time lag difference. Therefore, the sensor signals, located at different points in the harvester, were synchronized, by the time, with mass flow observed (measured by a load cell in the infield wagon).

The random sampling method was used to split the dataset into train and test data with a 70:30 ratio. The same ratio was used to carry out the training of each model used in this study. Nine combinations between the different sensors were evaluated (Table 2). The first combination consists of all eight points with the sensors on the harvester. One or more sensors were removed from the data set, and the new models were tested.

### 2.5. Analysis

The prediction error was calculated for each sampled point in the test data set, which is the difference between the predicted mass flow by the MLR and NARXs and observed mass flow. The metrics Root Mean Square Error (RMSE, Equation (7)) and Mean Absolute Percent Error (MAPE, Equation (8)) were used to evaluate and compare the models.

(7) $$\mathrm{RMSE}=\sqrt{\frac{1}{n}\sum_{i=1}^{n}{\left(y_{i}-x_{i}\right)}^{2}}$$

(8) $$\mathrm{MAPE}=\left[\frac{1}{n}\sum_{i=1}^{n}\frac{\left|y_{i}-x_{i}\right|}{x_{i}}\right]\times 100$$

where *n* is the number of the sample, *y_i_* is the predicted variable response, and *x_i_* is the observed variable response.

## 3. Results and Discussion

**Figure 4:**
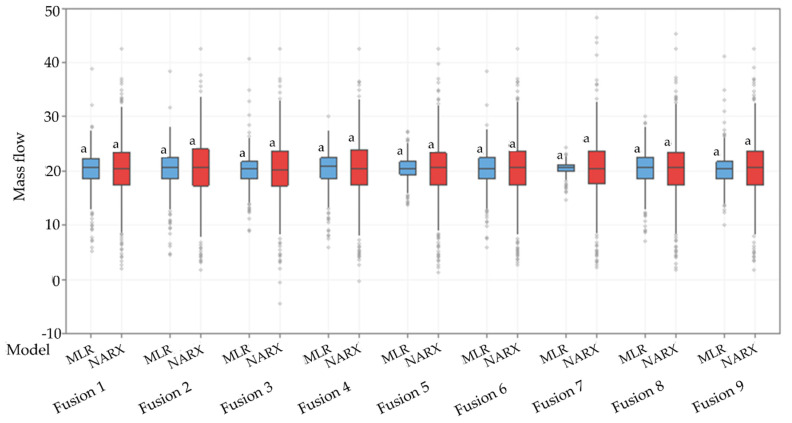
Boxplot of the mass flow (kg s^−1^) predicted by the models. The same letters above the box indicate no significant difference between the mean mass flow predicted value of the models (Tukey test; *p* < 0.05). MLR—multiple linear regression (blue box). NARX—nonlinear autoregressive networks with exogenous input (red box). The description of the different fusion models is in Table 2.

In the mean comparison analysis, there was no significant difference in the mean of the values predicted by the different models tested (Figure 4). However, the predictions using the NARX had a larger range of values than the predictions using MLR. That is, the MLR reduced the variability of the predicted data. This is a first indication that the NARX is each able to identify the mass flow variation within the harvester It is common for mass flow variation peaks to occur over short periods, since sugarcane has high yield variability over short distances within the row. In this way, the MLR may not be identifying these variations, which leads to a concentration of data close to the mean, as shown in the boxplot (Figure 4). The MLR with only the potentiometric displacement sensor in the feed roller (Fusion 5), and model with only a strain gauge sensor (Fusion 7), has the lower amplitude of the estimated data. On the other hand, there was no reduction in the amplitude of this data using the NARX.

All fusion models using NARX had a high correlation between the predicted and observed mass flow (Table 3). The best model was with all variables with input to the NARX, with a correlation of 0.99. The model with only the strain gauge sensors has a correlation of 0.17 with observed values, a lower value than other combinations of variables using the NARX. Using the MLR, the best correlation was found (*r* = 0.63), using all sensors as input variables of the model (Table 2). The removal of the input variables RBSG and LBSG did not affect the estimation of the mass flow by the MLR, presenting a moderate correlation with the observed mass flow (*r* = 0.62). There was no correlation of predicted values with that observed (*r* = 0.17) using only data from the four locations with strain gauge sensors as input variables in the MLR. A similar result was obtained using only the displacement of the feeder rollers 1 and 2 as MLR input (*r* = 0.32).

Mass flow prediction with the NARX showed a significant superiority over MLR (Table 3). NARX with all input variables had an RMSE of 0.3 kg s^−1^, representing an MAPE of 0.7%. The RMSE of the MLR was 240% higher than the NARX. In previous studies using parameters of the harvester engine [14], the ANN model performed better (MAPE = 5.6%) than the MLR (MAPE = 7.8%) to predict sugarcane mass over the collection time. The error of the prediction by NARX shows that this approach can be an alternative for predicting sugarcane yield in a harvester. This approach, using a new multi-sensor model, has similar or even superior performance to the approach using a single sensor or system for measuring sugarcane yield, such as using fiber optics [10], load cells [9], or a deflector plate [11]. However, although the NARX model with multi-sensors had a smaller error than the other approaches. A disadvantage with the other approaches for yield prediction is the use of multi-sensors, which can increase the cost of implementing this system.

Other studies comparing prediction models have also shown higher prediction accuracy using an NARX compared to an MLR [41,42]. According to some studies, the NARX was the best performing model compared with the MLR due to its ability to dynamically adapt its trajectory based on previous errors [43,44]. Using the values previously predicted by the NARX minimizes the current prediction error, while MLR does not have the dynamic ability to learn from the error values of previous predictions. The ability of a model to self-calibrate is important when it comes to sensing crops with high yield variability, such as sugarcane [45]. This high variability over small distances directly influences the feeding rate of the harvester during the sugarcane harvest and, consequently, there will be high mass flow variation over a short time interval.

The removal of strain gauge sensors from the elevator base (RBSG and LBSG) did not influence mass flow prediction. The NARX had an RMSE of 0.4 kg s^−1^ without these sensors, a tiny difference (0.1 kg s^−1^) to the model with these two sensors. The vibrations caused by the displacement of the conveyor during the transport of the billets, together with the vibrations resulting from the harvester displacement, may interfere with the strain gauge signals. On the other hand, this can explain the low performance (compared to the other models tested in this study) of the NARX and MLR in predicting the mass flow using only the four points instrumented with a strain gauge. MLR with the input variables RCSG, LCSG, RBSG, and LBSG has the worst performance of the models tested in this study. The lower deformation sensitivity of the base material related to the sugarcane mass in the elevator is a factor that may be related to low performance. That is, the mass contained in the elevator may be insufficient for the sensor-capable deformation at the base of the elevator.

Using only the variables EPS and CPS obtained excellent results using the NARX, with RMSE values of 0.4 kg s^−1^. RMSE equal to the previous model without the variables RBSG and LBSG, but with a higher MAPE value (1.6%). This shows that the power required in the chopper hydraulic system [46] and the elevator conveyor are directly correlated with the sugarcane mass flow. A previous study [47] has shown that the feeding rate is one significant variable affecting chopping quality. In this way, the increase in the chopping quality can be with the automation of chopper roller speed utilizing the accurate mass flow prediction by the NARX. The mass flow can also be used to increase the power demand efficiency during sugarcane chopping. The chopper system is the one that demands the highest potency in the sugarcane harvester [48], and the automation of the chopper system using mass flow predicted can significantly increase the harvester efficiency.

**Figure 5:**
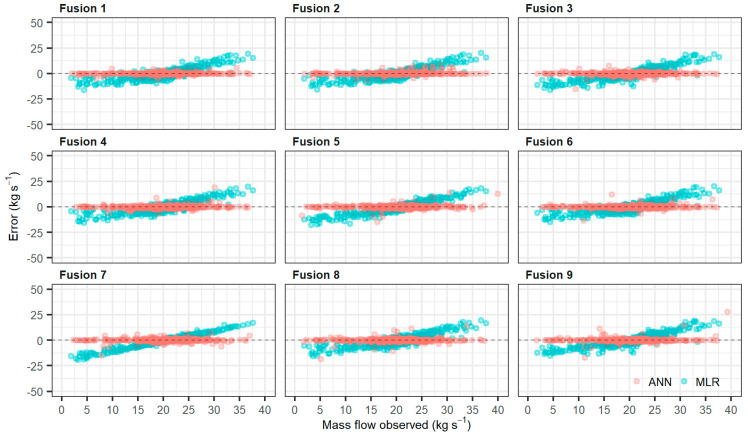
Scatter plot of the Multiple Linear Regression (MLR) and NARX Neural Network (ANN) models prediction error.

MLR decreases the estimated mass flow variability in the sugarcane harvester (Figure 5). With MLR, the error was higher in the extreme values. It decreased close to the mean value, increasing the linear trend, with results similar to the use of engine parameters as data input of the MLR on sugarcane mass prediction [14]. The MLR overestimated the lower mass flow observed (errors closed to 19.2 kg s^−1^) and underestimated the high mass flow observed with errors of 20.4 kg s^−1^. The smoothing of mass flow data leads to loss of detail of the variability of essential information within the context of PA, especially for decision-making and management of variability of sugarcane fields. Knowing the sugarcane yield at small spatial distances is fundamental to the site-specific investigations in sugarcane rows [45]. Erroneous mass flow values can influence decision-making during the automation of the harvester’s control processes, reducing machine efficiency.

NARX has lower dispersion error than MLR (Figure 5). The nonlinear model has a lower error dispersion than the MLR because of its ability to explain more response variability [32]. Moreover, the nonlinear method performs a better job at re-calibration, as NARX is better at prediction in the short term. Its descending gradient learning tends to become more effective due to its integrated memory that provides a smaller path for propagating the information gradient when the network is opened, instead of back-propagating the error signal [49].

**Figure 6:**
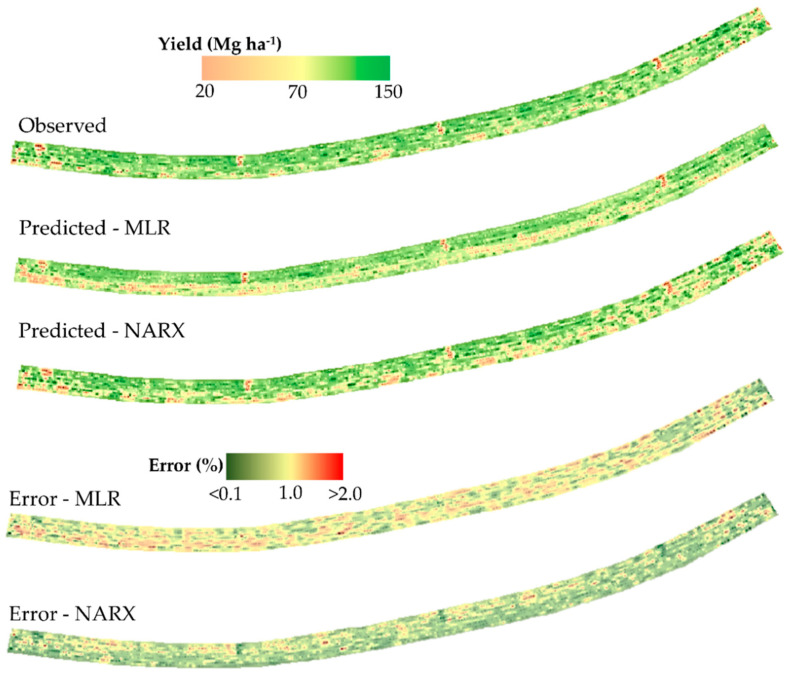
Yield map of the observed data predicted by multiple linear regression (MLR) and nonlinear autoregressive networks with exogenous input (NARX). Below the yield map contains the prediction error (%), spatialized.

Using the validation data it was possible to spatially analyze the performance of the MLR and NARX models (Figure 6). Using MLR and NARX with all sensors (Fusion 1, Table 2), which had the best result in the previous tests (Table 3), it was possible to obtain prediction errors under 2%. It was evident that the MLR underestimated the yield within the field, with the predicted error upper of 1%, while the data predicted by NARX were similar to the observed data. However, it is probably less accurate in short-distance predictions, because there has been smoothing in the short-distance. Nevertheless, the model was able to capture larger-scale patterns of spatial variability, which is suitable for AP applications. The spatial variability found in the yield map can be used to identify patterns with high yield potential or patterns with low yield potential where yields are unlikely to increase.

From this study, a harvester instrumented with multi-sensors to predict mass flow with the NARX does not require different calibrations in the field. However, a study with multiple harvesters within the same field is necessary. Multi-harvesters with different calibration settings are typical in agricultural fields; these cause discontinuity of the data values within the field, and require post-processing of the data to remove this calibration difference [50,51]. Overall, the approaches in this study help support different applications in PA. The data sensors and the processing needed to generate the mass flow data can be easily embedded and implemented in a sugarcane harvester to develop its automation in the adjustments over the operation time, facilitate adoption, and promote site-specific management of sugarcane fields.

## 4. Conclusions

The fusion of sensor signals improves the accuracy of mass flow prediction. The nonlinear autoregressive networks with exogenous input (NARX) may be a viable alternative to conventional regression models for predicting mass flow from this study. It generates accurate mass flow data without the need to post-process the data. These data provide accurate information essential for the automation of the control processes in the harvester. The sugarcane mass flow approach with multiple sensors is a potential approach to replace conventional yield monitors. The system generates accurate data with high sample density within sugarcane rows. With a GNSS, it will be possible to spatialize the data within the field, generating spatialized yield information, which is the most valuable information for the localized management of sugarcane fields.

## Figures and Tables

**Table 1:** Amount of data used for training, testing, and validation of the models in this study.

Row Number	Train and Test	Validation
1	389	69
2	358	63
3	301	53
4	309	55
5	351	62
6	331	58
7	344	61
8	332	59
9	343	60

**Table 2:** Combinations between the different sensors tested in this study.

Models Input Variables	Sensor
Fusion 1 = {CPS, EPS, FRO1, FRO2, RCSG, LCSG, RBSG, LBSG}	PS, SGS, DPS
Fusion 2 = {CPS, EPS, FRO1, FRO2, RCSG, LCSG}	PS, SGS, DPS
Fusion 3 = {EPS, RCSG, LCSG}	PS, SGS
Fusion 4 = {CPS, FRO1, FRO2}	PS, DPS
Fusion 5 = {FRO1, FRO2}	DPS
Fusion 6 = {CPS, EPS}	PS
Fusion 7 = {RCSG, LCSG, RBSG, LBSG}	SGS
Fusion 8 = {EPS}	PS
Fusion 9 = {CPS}	PS

PS: Pressure sensor. SGS: Strain gauge sensor. PDS: Potentiometric displacement sensor. LBSG: SGS in the left base. RBSG: SGS in the right base. LCSG: SGS in the right hydraulic cylinder. RCSG: SGS in the left hydraulic cylinder. FRO1: PDS in the feeder roll #1. FRO2: PDS in the feeder roll #2. CPS: PS in the chopper hydraulic motor. EPS: PS in the conveyor hydraulic motor.

**Table 3:** Mass flow prediction assessment metrics of the models tested in this study.

Models Input Variables	Correlation		RMSE (kg s^−1^)		MAPE (%)	
	MLR	NARX	MLR	NARX	MLR	NARX
CPS, EPS, FRO1, FRO2, RCSG, LCSG, RBSG, LBSG	0.63	0.99	1.6	0.3	4.9	0.7
CPS, EPS, FRO1, FRO2, RCSG, LCSG	0.62	0.98	1.6	0.4	4.9	0.8
EPS, RCSG, LCSG	0.56	0.96	1.7	0.5	6.3	2.4
CPS, FRO1, FRO2	0.50	0.97	1.8	0.5	5.4	1.3
FRO1, FRO2	0.32	0.96	1.9	0.5	7.3	1.0
CPS, EPS	0.61	0.97	1.6	0.4	5.1	1.6
RCSG, LCSG, RBSG, LBSG	0.17	0.93	2.0	0.7	8.8	1.2
EPS	0.49	0.95	1.8	0.6	5.7	1.2
CPS	0.56	0.94	1.7	0.6	6.5	2.5

LBSG: strain gauge in the left base. RBSG: strain gauge in the right base. LCSG: strain gauge in the right hydraulic cylinder. RCSG: strain gauge in the left hydraulic cylinder. FRO1: potentiometric sensor in the feeder roll #1. FRO2: potentiometric sensor in the feeder rolls #2. CPS: pressure sensor in the chopper hydraulic motor. EPS: pressure sensor in the conveyor hydraulic motor.

## Data Availability

Not applicable.
